# A CT-based radiomics model for preoperative prediction of lymphovascular invasion in colorectal cancer

**DOI:** 10.3389/fonc.2026.1811229

**Published:** 2026-04-24

**Authors:** Yingcheng Bai, Long Li, Chunhong Xu, Jinghui Zhang, Siyuan Jiang, Jie Wang, Suxia Qi

**Affiliations:** 1 Department of General Surgery, No.971 Hospital of the People’s Liberation Army Navy, Qingdao, China; 2 Department of Endocrinology, No.971 Hospital of the People’s Liberation Army Navy, Qingdao, China; 3 No.971 Hospital of the People's Liberation Army Navy, Qingdao, China; 4 Department of Gastroenterology, Qingdao Hospital, University of Health and Rehabilitation Sciences (Qingdao Municipal Hospital), Qingdao, China

**Keywords:** colorectal cancer, lymphovascular invasion, predictive model, preoperative CT, radiomics

## Abstract

**Objective:**

This study aimed to develop and validate a computed tomography (CT)-based radiomics nomogram for the preoperative prediction of lymphovascular invasion (LVI) in colorectal cancer (CRC).

**Methods:**

In this retrospective study, CRC patients were randomly partitioned into training and validation sets at a 7:3 ratio, and were classified as LVI-positive or LVI-negative based on postoperative histopathology. In the training set, univariate analysis, Least Absolute Shrinkage and Selection Operator (LASSO), and multivariate Logistic regression analysis were used to identify the influencing factors of LVI. A combined nomogram integrating the predictors was developed. Model discrimination was evaluated using the area under the receiver operating characteristic curve (AUC). Calibration and clinical utility were assessed with calibration curves and decision curve analysis (DCA), respectively.

**Results:**

Among the 252 patients in the training set, 70 (27.78%) were LVI-positive. In the validation set of 108 patients, 31 (28.70%) were LVI-positive. Multivariate analysis identified the tumor volume, maximum tumor diameter, depth of tumor invasion, maximum short-axis diameter of regional lymph nodes, Carcinoembryonic Antigen level, Neutrophil-to-Lymphocyte Ratio (NLR), standard deviation of CT value of tumor parenchyma, and Rad-score as independent predictors of LVI (P<0.05). The nomogram incorporating these factors demonstrated good predictive performance, with an AUC of 0.776 (95% CI: 0.694-0.858) in the training set and 0.722 (95% CI: 0.575-0.869) in the validation set. Calibration curves indicated satisfactory agreement between predictions and observations. DCA confirmed the clinical net benefit of the nomogram across a wide range of risk thresholds.

**Conclusion:**

The proposed CT-based radiomics nomogram, featuring the Rad-score, NLR, and intratumoral heterogeneity, provides a non-invasive and reliable tool for preoperative LVI prediction in CRC. This model holds promise for enhancing individualized clinical strategy and prognosis assessment.

## Introduction

Colorectal cancer (CRC) is a malignancy with a leading global incidence and mortality rate, and its diagnosis and treatment paradigm is rapidly evolving towards precision and individualization (1, 2). Lymphovascular invasion (LVI), defined as the invasion of tumor cells into the endothelial-lined lumina or walls of lymphatic or blood vessels, is a critical pathological and biological characteristic of CRC. It serves as a core indicator for assessing the invasive and metastatic potential of the tumor, guiding decisions on postoperative adjuvant therapy, and evaluating patient prognosis (3–5). Currently, definitive diagnosis of LVI relies on postoperative histopathological examination and cannot directly inform preoperative clinical decision-making.

Radiomics, an emerging interdisciplinary technology, enables the extraction and quantitative analysis of high-throughput features from medical images. This approach can uncover microscopic pathophysiological information beyond visual perception, reflecting tumor biology such as cellular density, angiogenesis, and heterogeneity, thereby providing a novel technical pathway for the precise preoperative assessment of tumors (6, 7). In recent years, radiomics has become a research focus for the preoperative prediction of CRC-related pathological features, with applications across various imaging modalities offering diverse strategies for LVI prediction. Zhang et al. developed a clinical-radiomics model based on multi-parametric magnetic resonance imaging (MRI). By extracting 3356 radiomics features and incorporating MRI-reported extramural vascular invasion status, their model aimed to jointly predict LVI and perineural invasion (PNI). However, this model relies on MRI and did not fully address the insufficient sensitivity in predicting small-vessel invasion (8). Yang et al. constructed a model based on positron emission tomography-computed tomography (PET-CT) radiomics features combined with SUVmean, maximum tumor diameter, and lymph node metastasis status. Nonetheless, PET-CT has limitations such as higher radiation dose and cost, making it unsuitable for routine preoperative assessment (9). The team of Li et al. developed a combined model based on CT radiomics, incorporating features from both the tumor and peritumoral regions. However, their feature selection process did not sufficiently integrate common clinical laboratory indicators, leaving room for improvement in the predictive efficacy for early-stage LVI (10).

Consequently, this study retrospectively enrolled CRC patients. Radiomics features were extracted from preoperative contrast-enhanced abdominal CT images in the portal venous phase. Key features were selected using methods such as Least Absolute Shrinkage and Selection Operator (LASSO) regression to construct a radiomics score (Rad-score). Subsequently, a combined clinical-radiomics prediction model was established by integrating Rad-score with clinical risk factors. The model’s predictive performance, calibration, and clinical net benefit were validated using both training and validation sets. The objective is to provide an accurate and practical preoperative tool for predicting LVI, thereby offering a scientific basis for individualized treatment decisions and prognostic assessment in CRC patients.

## Methods

### Study population and sample size estimation

Based on previous similar studies (8–10) and using an estimated LVI-positive rate of 30% in CRC patients as a benchmark, the sample size was calculated following prediction model development guidelines. According to the “events per variable” (EPV) principle, a minimum of 10 outcome events per predictor variable is recommended to ensure model stability. Based on the anticipated number of predictors in the final multivariate model (approximately 8–10 variables), at least 80–100 LVI-positive events were required. With an estimated LVI-positive rate of 30%, this corresponds to a minimum total sample size of approximately 267–333 cases. Considering a 15% allowance for potential missing data, the required sample size was estimated to be at least 307–383 cases. This study planned to enroll 360 CRC patients, meeting this requirement. Patients were randomly divided into a training set (n=252) and a validation set (n=108) in a 7:3 ratio.

### Inclusion and exclusion criteria

Inclusion criteria: (1) Pathologically confirmed CRC following surgery, with diagnosis adhering to the ACG Clinical Guidelines: Colorectal Cancer Screening 2021 (11), confirming tumor origin from the mucosal epithelium of the colon or rectum. (2) Completion of contrast-enhanced abdominal CT scanning including portal venous phase images within 1–2 weeks prior to surgery, ensuring the feasibility of extracting high-quality radiomics features. (3) Availability of a postoperative pathological report explicitly documenting LVI status (positive/negative), where the presence of tumor cells within endothelial-lined spaces or destroying the lymphatic/vascular wall constituted a positive finding. (4) Complete and accessible clinical, imaging, and pathological data.

Exclusion criteria: (1) Receipt of neoadjuvant radiotherapy, chemotherapy, targeted therapy, or other anti-tumor treatments prior to surgery, to avoid interference with tumor imaging characteristics and pathological status. (2) Presence of severe artifacts, incomplete sequences, or insufficient resolution in CT images, precluding clear delineation of the region of interest (ROI). (3) Missing key clinical or pathological data that would affect variable screening and model construction. (4) Coexistence of other malignancies or severe systemic diseases that might confound the study outcome. (5) Primary tumor with a maximum diameter of<5 mm, as radiomics feature extraction from such small lesions is limited.

### Data collection

Clinical Data Collection: Demographic, tumor-related, and laboratory indicators were collected from the electronic medical record system, preoperative examination records, and pathology reports. These included age, sex, body mass index (BMI), smoking history, alcohol consumption history, tumor location, CT-reported T stage (CT-T), CT-reported N stage (CT-N), histological grade (well, moderately, poorly differentiated), tumor volume, maximum tumor diameter, distance from the anal verge, depth of tumor invasion, maximum short-axis diameter of regional lymph nodes, carcinoembryonic antigen (CEA), carbohydrate antigen 19-9 (CA19-9), carbohydrate antigen 125 (CA125), neutrophil-to-lymphocyte ratio (NLR), and platelet-to-lymphocyte ratio (PLR). All clinical data were independently extracted by two trained clinicians and cross-checked. The inter-rater agreement yielded a Kappa value >0.85 for categorical variables and an intraclass correlation coefficient (ICC) >0.90 for continuous variables.

Imaging Data Collection: Preoperative imaging was performed using contrast-enhanced abdominal CT, which necessarily included the portal venous phase. Scanning parameters were set as follows: tube voltage 120 kV, tube current using automatic milliampere-second technique, slice thickness ≤1.5 mm, slice interval ≤1 mm. A non-ionic contrast agent was injected via an antecubital vein at a rate of 3-3.5 mL/s, with a portal venous phase scan delay of 50–70 seconds.

Medical imaging analysis software and the PyRadiomics package were used for post-processing. Two board-certified radiologists, blinded to clinical and pathological data, independently delineated the three-dimensional ROI on the portal venous phase images using 3D Slicer 5.4.0 (open-source medical image analysis software). The ROI encompassed the entire tumor parenchyma and a 5-mm peritumoral region, while avoiding areas of necrosis, calcification, and major blood vessels. This ROI delineation strategy was formulated based on the universal biological characteristics of tumors and the technical requirements of radiomic feature extraction. The 5-mm peritumoral region was included because it constitutes the key microenvironment for tumor invasion and lymphovascular spread, and its radiomic features can reflect the tumor invasive potential associated with LVI. Necrotic and calcified areas within the tumor were excluded because these regions contain no viable tumor cells, lack specific imaging features reflecting the biological behavior of tumor invasion and metastasis, and would introduce non-specific image noise that interferes with the accuracy of radiomic feature extraction and the predictive efficiency of the model (13, 14). The tumor volume (cm³) was automatically calculated by the medical imaging analysis software based on the delineated three-dimensional ROI, which was one of the key tumor-related imaging parameters measured in this study. Tumor-related imaging parameters were measured. Subsequently, 2107 radiomics features were extracted, including first-order statistics, shape-based features, and texture features. Prior to feature extraction, images underwent preprocessing steps such as resampling and filtering, followed by feature standardization using the Z-score method. Based on the selected stable and discriminative key features, corresponding regression coefficients derived from logistic regression analysis were used to calculate the Rad-score (10). Radiomics features were first subjected to dimension reduction using the LASSO regression method, with 10-fold cross-validation applied to determine the optimal penalty parameter (λ). After feature selection, multivariate logistic regression analysis was performed to derive the regression coefficients for the remaining key features. The Rad-score was then calculated using the formula: $Rad-score\;=\;\Sigma \;\left(Coef_{i}\;\times \;F_{i}\right)$, where Coef_i_ denotes the regression coefficient of the i-th key radiomics feature (F_i_). The key features included in the Rad-score and their corresponding coefficients were as follows: Feature 1: 0.82, Feature 2: -0.57, Feature 3: 0.41, Feature 4: -0.33, Feature 5: 0.29. All imaging measurements and feature extractions were performed independently by the two radiologists, with an ICC >0.90 ensuring reliability. Discrepancies were resolved through consultation with a third senior radiologist.

### Outcome definition

The primary outcome of this study was the LVI status of CRC patients, categorized as LVI-positive or LVI-negative. LVI status was determined according to the European guidelines for quality assurance in colorectal cancer screening and diagnosis (First Edition) (12). LVI-positive was defined by the observation of tumor cells within an endothelial-lined space (lymphatic or blood vessel lumen) or tumor cells destructively invading the vessel wall on pathological slides. The absence of such findings was defined as LVI-negative. To ensure reliability, all pathological slides were independently and blindly assessed by two pathologists, each with over five years of experience in gastrointestinal tumor pathology. In cases of disagreement, a consensus was reached through joint review. The final inter-observer agreement yielded a Kappa value >0.85.

### Statistical analysis

Statistical analyses were performed using SPSS 26.0 and R 4.5.1 software. Graphs were generated with GraphPad Prism 9.0. A two-sided P value<0.05 was considered statistically significant.

Continuous variables were tested for normality. Those with a normal distribution are presented as mean ± standard deviation and compared using the independent samples t-test. Non-normally distributed data are presented as median (interquartile range) and compared using the Mann-Whitney U test. Categorical variables are presented as numbers (percentages) and compared using the chi-square test or Fisher’s exact test. Patients were randomly divided into training and validation sets in a 7:3 ratio using a random number table. The comparability of baseline clinical characteristics between the two sets was verified using the aforementioned tests. In the training set, univariate analysis was performed with LVI status as the binary dependent variable and various factors as independent variables. Potential predictors with P< 0.05 were selected for further analysis. LASSO regression was employed for variable compression and redundancy elimination using the glmnet package in R 4.5.1; the optimal tuning parameter (λ) was determined via 10-fold cross-validation based on the lambda.1se criterion to identify core predictive variables with the strongest discriminative ability. Then these variables were incorporated into a multivariable logistic regression model using the glm function in R 4.5.1, where stepwise backward selection (likelihood ratio test, P = 0.05 as the cut-off value) was used to finalize the independent predictors, and calculating odds ratios (OR) and their 95% confidence intervals (CI). Variance inflation factors (VIF) were calculated to exclude multicollinearity (VIF threshold<10). A nomogram was constructed using the rms package in R. The model was interpreted using SHapley Additive exPlanations (SHAP) values to quantify the contribution of each feature to the prediction outcome, and feature importance plots and dependence plots were generated. The discriminative ability of the model was evaluated by plotting receiver operating characteristic (ROC) curves and calculating the area under the ROC curve (AUC) with its 95% CI. Calibration curves were plotted, and the Hosmer-Lemeshow test was used to assess the agreement between predicted probabilities and actual outcomes. Internal validation was performed using 1000 bootstrap resamples to correct for overfitting bias. Decision curve analysis (DCA) was used to evaluate the clinical application value of the nomogram by calculating the net benefit at different threshold probabilities.

## Results

### Comparison of baseline characteristics between training and validation sets

A total of 360 CRC patients were enrolled. Among the 252 patients in the training set, 70 (27.78%) were LVI-positive. In the validation set of 108 patients, 31 (28.70%) were LVI-positive. No statistically significant differences were observed in the baseline characteristics between the training and validation sets (all *P*>0.05) (**Table 1**).

**Table 1:** Comparison of baseline characteristics between training and validation sets.

Variable		Training set (n=252)	Validation set (n=108)	*t/χ²*	*P*
Age (years)		57.26 ± 7.97	56.54 ± 6.57	0.826	0.409
Gender	Male	142(56.35)	55(50.93)	0.897	0.344
	Female	110(43.65)	53(49.07)		
BMI (kg/m²)		23.71 ± 2.32	23.57 ± 3.22	0.464	0.643
Smoking	Yes	56(22.22)	30(27.78)	1.195	0.274
	No	196(77.78)	78(72.22)		
Alcohol Drinking	Yes	42(16.67)	25(23.15)	2.097	0.148
	No	210(83.33)	83(76.85)		
Tumor Location	Colon	108(42.86)	40(37.04)	1.058	0.304
	Rectum	144(57.14)	68(62.96)		
CT-T	T1-2	47(18.65)	25(23.15)	1.292	0.524
	T3	155(61.51)	60(55.56)		
	T4	50(19.84)	23(21.30)		
CT-N	N0	105(41.67)	41(37.96)	1.169	0.557
	N1	105(41.67)	44(40.74)		
	N2	42(16.67)	23(21.30)		
Histological Grade	Well-differentiated	33(13.09)	14(12.96)	0.801	0.670
	Moderately-differentiated	160(63.49)	64(59.26)		
	Poorly-differentiated	59(23.41)	30(27.78)		
Tumor Volume (cm³)		9.09 ± 3.65	9.25 ± 4.05	0.369	0.713
Maximum Tumor Diameter (mm)		33.18 ± 7.83	34.02 ± 6.88	0.966	0.335
Distance from Anal Verge (cm)		8.04 ± 3.79	7.85 ± 3.55	0.444	0.657
Tumor Invasion Depth (mm)		7.12 ± 3.43	6.65 ± 3.05	1.231	0.219
Maximum Short-Axis Diameter of Regional Lymph Nodes (mm)		10.46 ± 2.72	10.01 ± 3.21	1.361	0.174
CEA (ng/mL)		5.54 ± 2.72	5.21 ± 2.42	1.089	0.277
CA19-9 (U/mL)		12.65 ± 6.62	13.24 ± 7.32	0.750	0.454
CA125 (U/mL)		12.79 ± 4.51	13.22 ± 5.54	0.772	0.440
NLR		2.21 ± 0.97	2.34 ± 1.02	1.147	0.252
PLR		131.91 ± 30.55	137.25 ± 35.12	1.452	0.148
Mean CT Value of Tumor Parenchyma (HU)		41.90 ± 7.76	40.98 ± 7.47	1.042	0.298
Standard Deviation of CT Value of Tumor Parenchyma (HU)		9.13 ± 2.77	8.78 ± 3.01	1.070	0.285
Rad-score		0.45 ± 0.17	0.48 ± 0.18	1.507	0.133

### Univariate analysis of LVI in CRC patients

Univariate analysis showed significant differences between LVI-positive and LVI-negative groups in the training cohort for several variables, including tumor volume, maximum tumor diameter, depth of tumor invasion, maximum short-axis diameter of regional lymph nodes, CEA level, NLR, mean CT value of tumor parenchyma, standard deviation of CT value of tumor parenchyma, and Rad-score (all *P*< 0.05) (**Supplementary Table 1**).

### Multivariate logistic regression analysis of LVI in CRC patients

**Figure 1:**
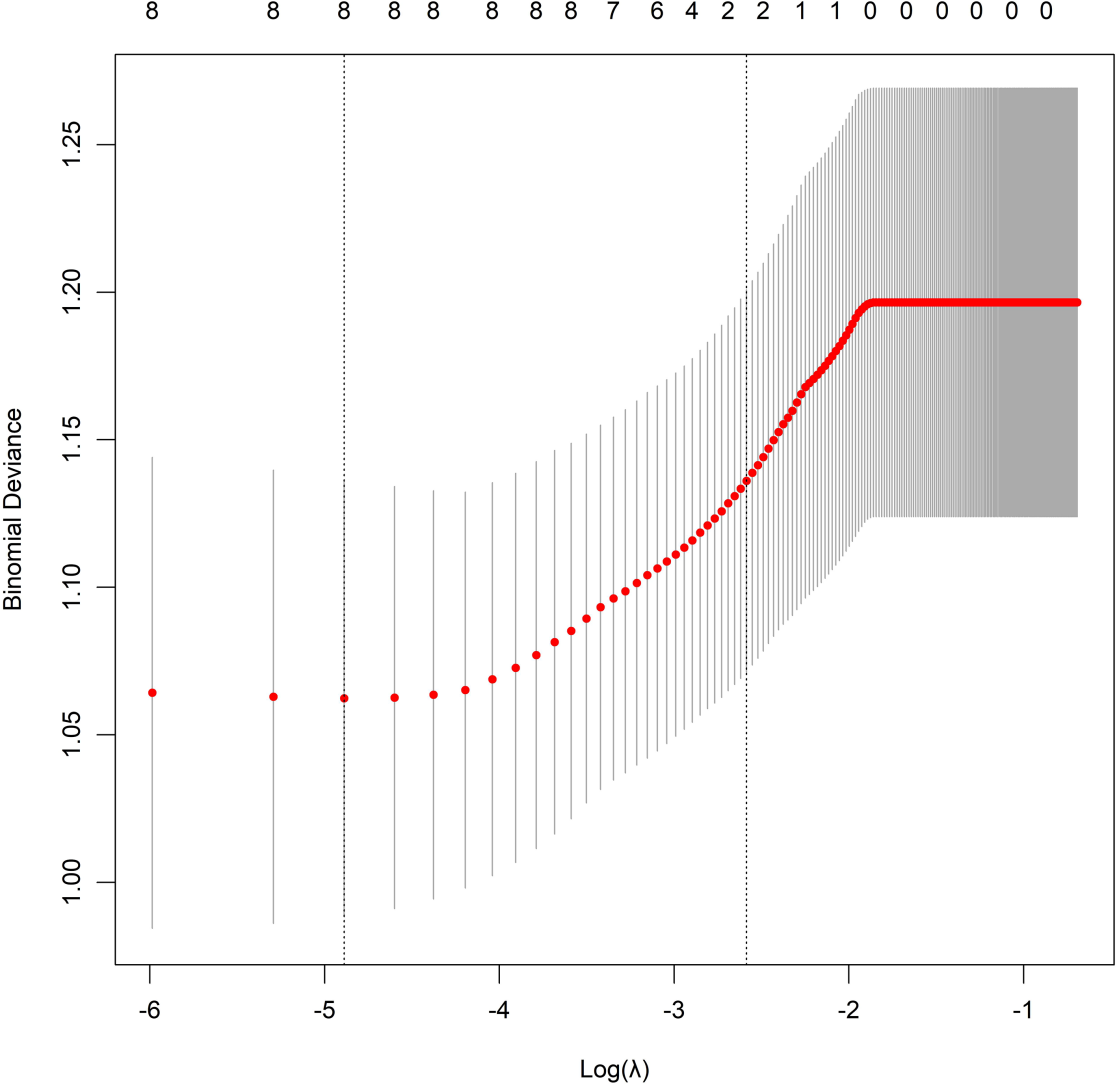
Least absolute shrinkage and selection operator regression variable selection.

LVI positivity was designated as the dependent variable (1 = positive group, 0 = negative group). Variables with *P*< 0.05 in the univariate analysis were included in a LASSO regression for variable selection, using the lambda.1se criterion (**Figure 1**). Subsequently, the optimally selected predictors were entered into a multivariate logistic regression model. The analysis identified tumor volume, maximum tumor diameter, depth of tumor invasion, maximum short-axis diameter of regional lymph nodes, CEA level, NLR, standard deviation of CT value of tumor parenchyma, and Rad-score as independent risk factors for LVI positivity (all *P*< 0.05) (**Table 2**). Multicollinearity diagnostics showed that the VIF for tumor volume, maximum tumor diameter, depth of tumor invasion, maximum short-axis diameter of regional lymph nodes, CEA, NLR, standard deviation of CT value of tumor parenchyma, and Rad-score were 1.035, 1.021, 1.028, 1.035, 1.039, 1.057, 1.051, and 1.064, respectively. These results indicate minimal multicollinearity among the variables, suggesting a low degree of linear correlation. Consequently, the adverse impact of multicollinearity on model estimation and inference during regression modeling and subsequent analyses is negligible, thereby ensuring the model’s stability and interpretability.

**Table 2:** Multivariate logistic regression analysis of LVI in CRC patients.

Factor	β	SE	Wald	*P*	OR	95%CI
Tumor Volume	0.086	0.043	4.053	0.044	1.090	1.002-1.185
Maximum Tumor Diameter	0.042	0.021	4.130	0.042	1.043	1.001-1.086
Tumor Invasion Depth	0.095	0.046	4.197	0.040	1.099	1.004-1.203
Maximum Short-Axis Diameter of Regional Lymph Nodes	0.115	0.058	3.875	0.049	1.122	1.001-1.257
CEA	0.132	0.053	6.186	0.013	1.141	1.028-1.266
NLR	0.369	0.163	5.131	0.024	1.447	1.051-1.991
Mean CT Value of Tumor Parenchyma	-0.011	0.021	0.257	0.612	0.989	0.950-1.031
Standard Deviation of CT Value of Tumor Parenchyma	0.140	0.057	5.987	0.014	1.150	1.028-1.286
Rad-score	0.704	0.200	12.396	0.001	2.022	1.366-2.993

### Construction of a nomogram prediction model for LVI in CRC patients

**Figure 2:**
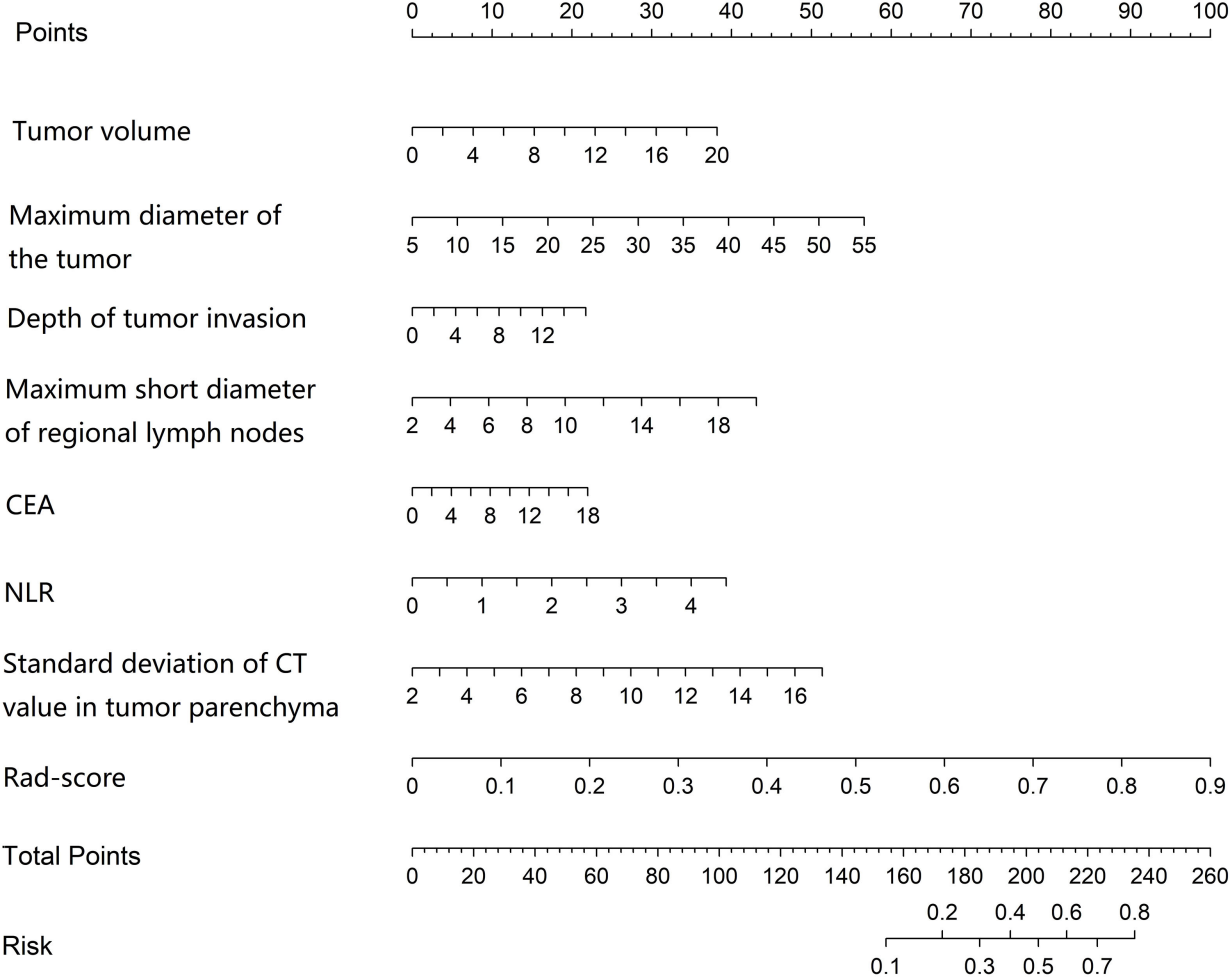
Nomogram for predicting lymphovascular invasion in colorectal cancer patients.

**Figure 3:**
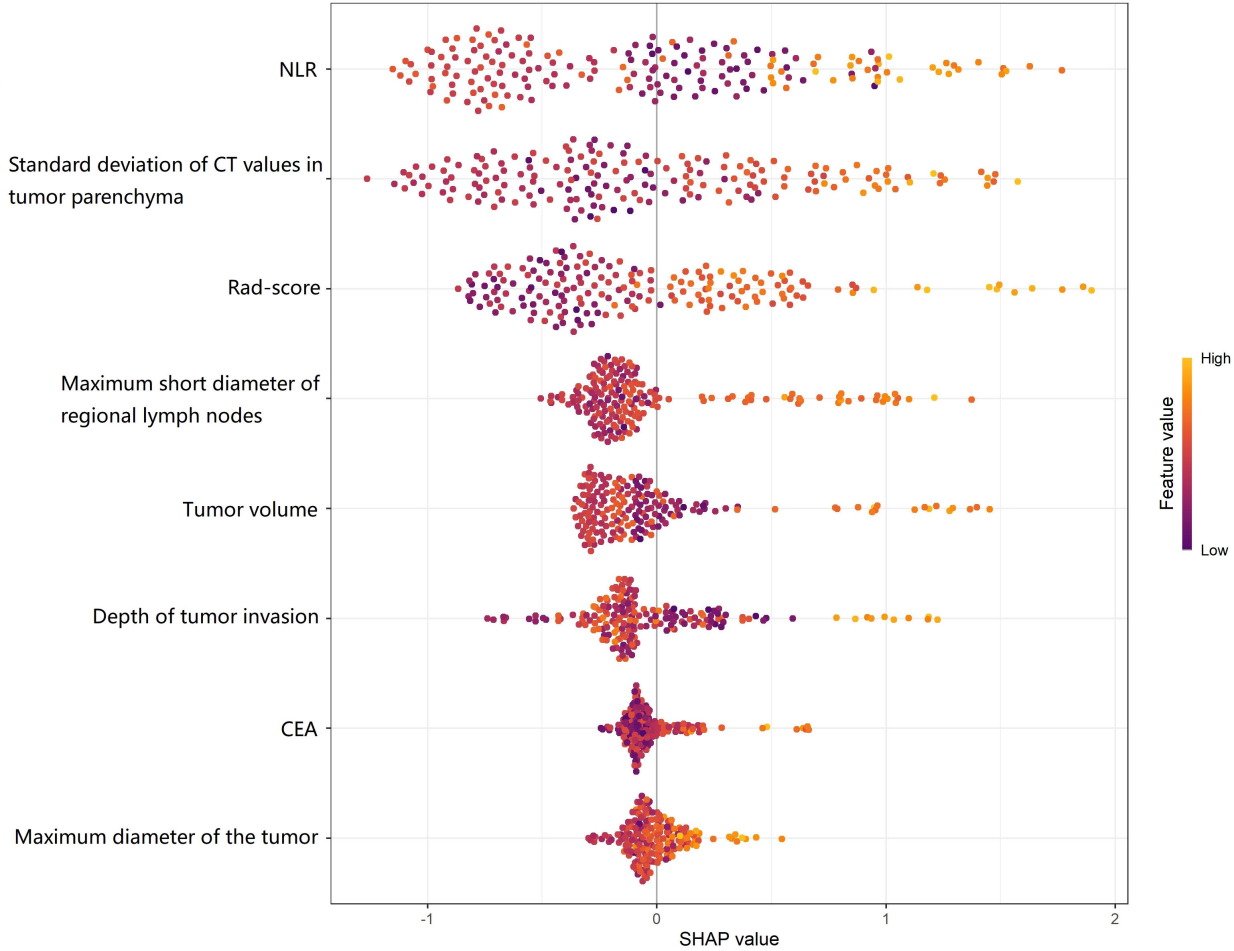
SHapley Additive exPlanations summary plot for variable importance.

Based on the independent risk factors identified by multivariate logistic regression analysis (including tumor volume, maximum tumor diameter, depth of tumor invasion, maximum short-axis diameter of regional lymph nodes, CEA level, NLR, standard deviation of CT value of tumor parenchyma, and Rad-score), a nomogram was constructed to predict the probability of LVI in CRC patients. Each independent risk factor in the model was assigned a corresponding point score. The total score, calculated by summing individual points, predicts the probability of LVI status in CRC patients, with a higher total score indicating greater predictive accuracy (**Figure 2**). The SHAP variable importance plot indicated that NLR, standard deviation of CT value of tumor parenchyma, and Rad-score had relatively greater influence on the model output (**Figure 3**).

### Evaluation and validation of nomogram prediction model for LVI in CRC patients

**Figure 4:**
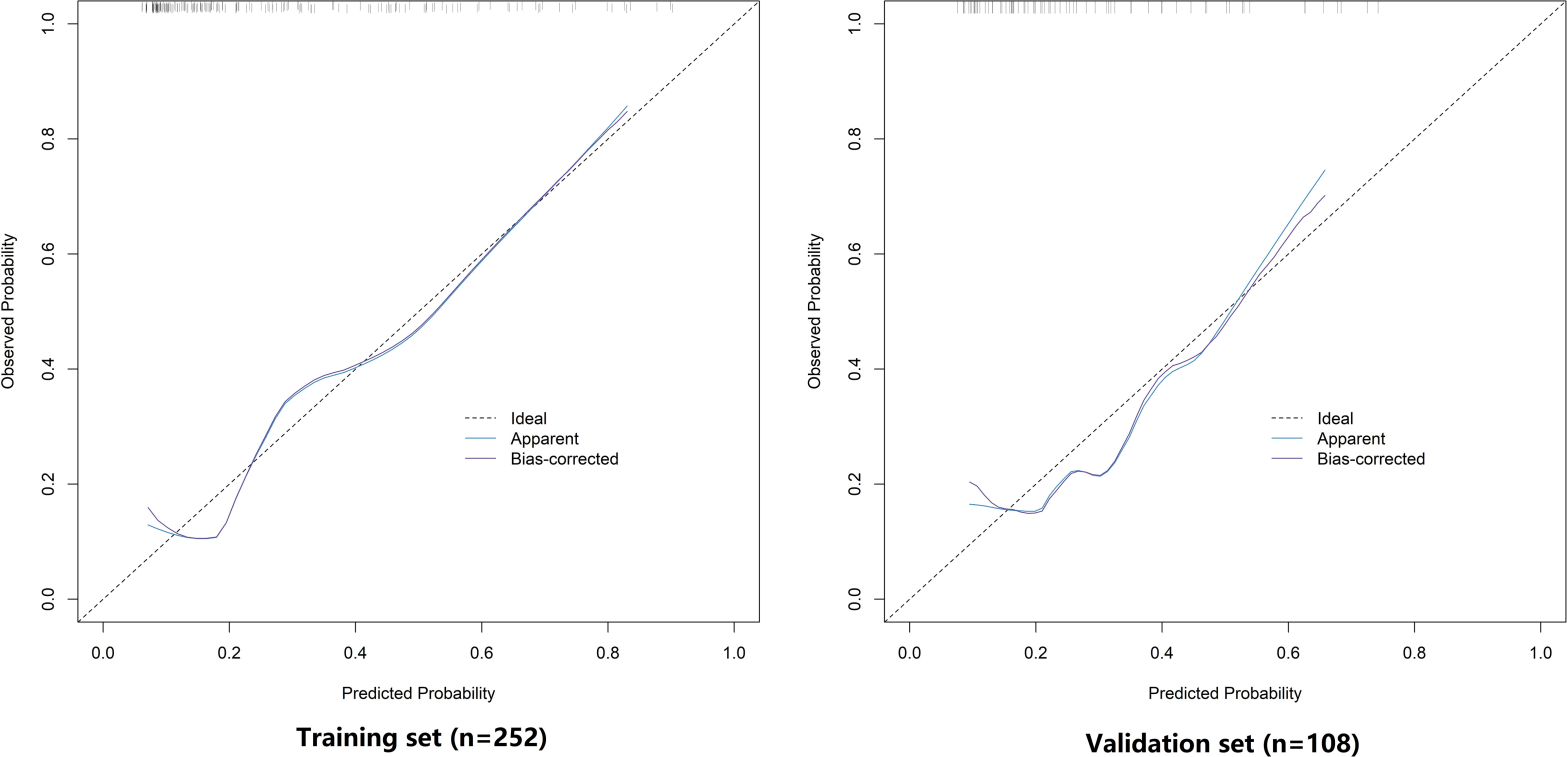
calibration curve analysis of the prediction model in the training and validation sets.

**Figure 5:**
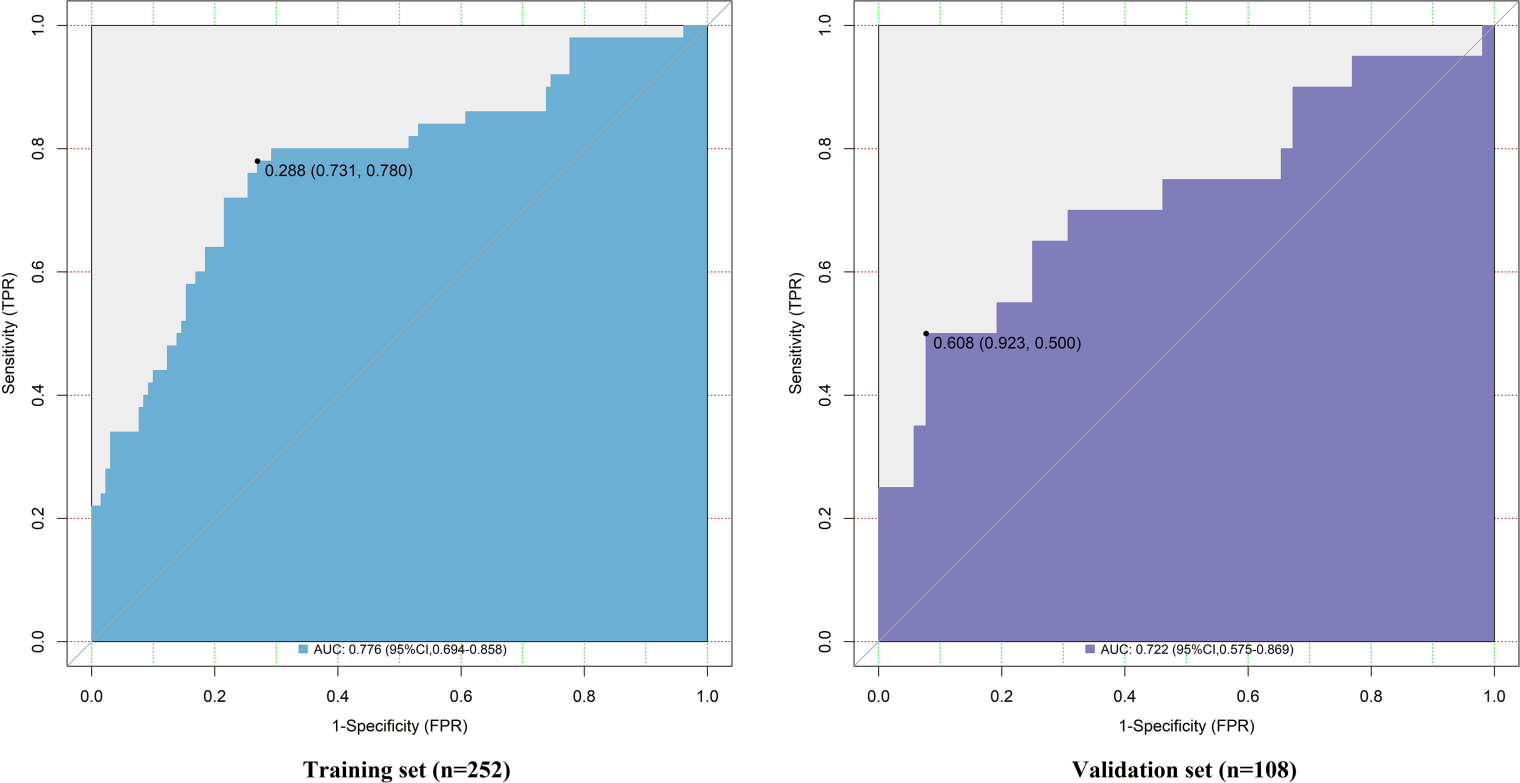
Receiver operating characteristic curves of the prediction model in the training and validation sets.

In the training and validation sets, the C-index of the nomogram model was 0.776 and 0.722, respectively. The calibration curves demonstrated mean absolute errors between predicted and observed probabilities of 0.153 and 0.180 for the training and validation sets, respectively (**Figure 4**). The Hosmer-Lemeshow test yielded χ² values of 7.865 (*P* = 0.447) and 4.960 (*P* = 0.762) for the two datasets, indicating good calibration. ROC curve analysis showed that the AUC for the nomogram model was 0.776 (95% CI: 0.694–0.858) in the training set and 0.722 (95% CI: 0.575–0.869) in the validation set (**Figure 5**). The sensitivity and specificity were 0.780 and 0.731 for the training set, and 0.750 and 0.538 for the validation set, respectively.

### Decision curve analysis of nomogram prediction model for LVI in CRC patients

**Figure 6:**
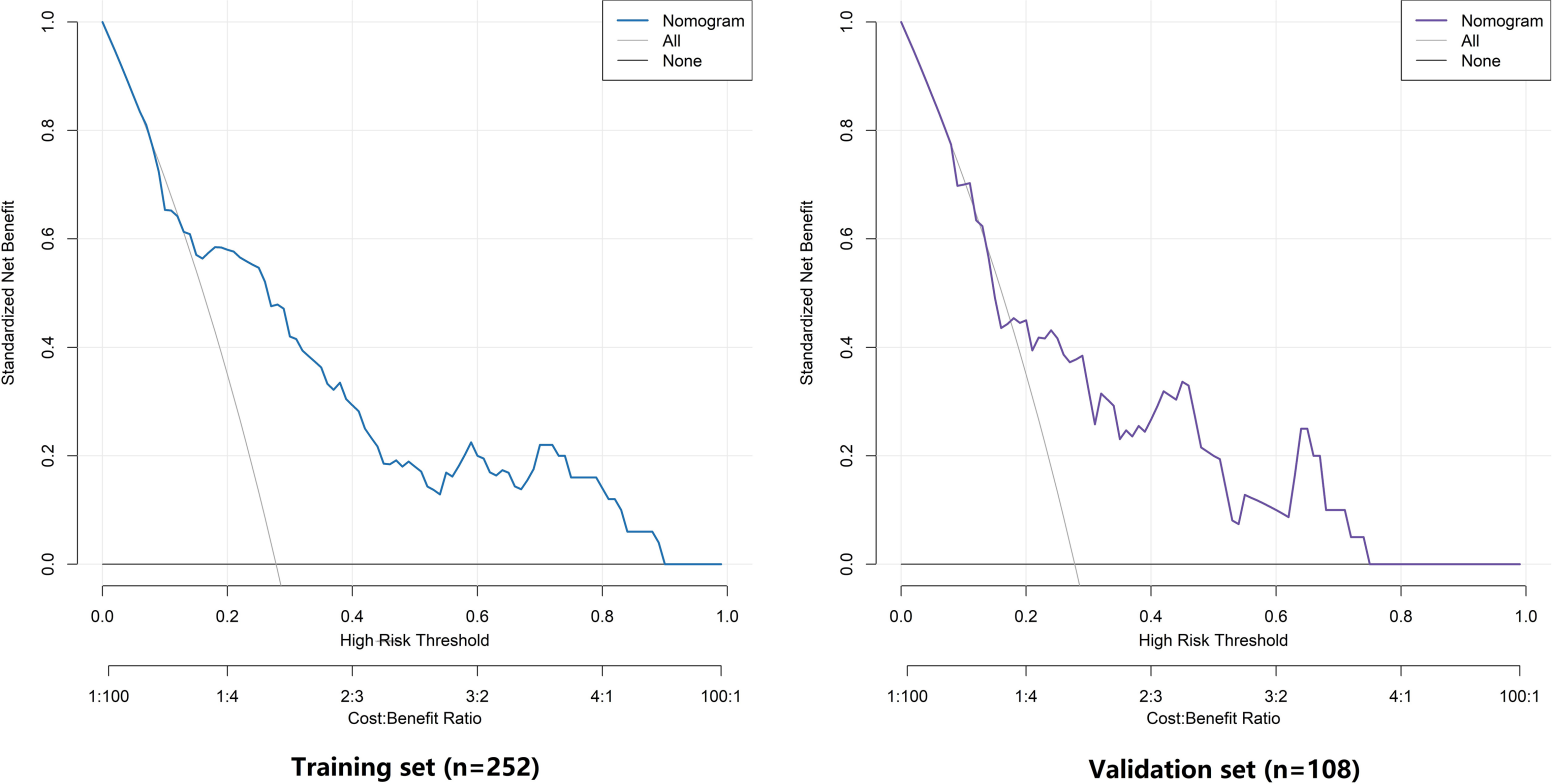
Decision curve analysis for the nomogram model in the training and validation sets.

DCA demonstrated that, within the high-risk threshold probability range of 0 to 0.85, the nomogram model in the training set provided a superior standardized net benefit compared to the treat-all or treat-none strategies, indicating its clinical utility during the training phase. In the validation set, the model maintained a net benefit advantage within the threshold range of 0 to 0.75. These findings suggest that the model not only possesses clinical decision-making value in the training phase but also exhibits robust generalizability in an independent validation cohort, reflecting reliable clinical practicality and potential for broader application (**Figure 6**).

### Comparison of predictive performance among different prediction models

To further verify the superiority of the constructed nomogram combined model and clarify its clinical application value, we conducted a comparative analysis of the predictive performance of three nomogram models (the combined model, Rad-score-only model, and clinical indicator-only model) in the training and validation sets. The results showed that the combined nomogram model had the highest AUC, sensitivity and specificity in both the training set and the validation set, which was significantly higher than the Rad-score-only model and the clinical indicator-only model (**Table 3**). Combined with the results of DCA, the nomogram combined model had a significant clinical net benefit in a wide range of risk thresholds. These findings indicated that the model had practical clinical application value despite the AUC of 0.722 in the validation set, and its predictive performance was improved compared with the single models.

**Table 3:** Comparison of predictive performance among different models for LVI in CRC.

Model	Dataset	AUC (95%CI)	Sensitivity	Specificity
Nomogram combined model	Training set	0.776 (0.694-0.858)	0.780	0.731
	Validation set	0.722 (0.575-0.869)	0.750	0.538
Rad-score-only model	Training set	0.692 (0.601-0.783)	0.652	0.689
	Validation set	0.655 (0.502-0.808)	0.613	0.597
Clinical indicator-only model	Training set	0.675 (0.581-0.769)	0.628	0.664
	Validation set	0.638 (0.480-0.796)	0.581	0.562

## Discussion

This study retrospectively enrolled 360 CRC patients. Based on preoperative contrast-enhanced abdominal CT images from the portal venous phase, radiomics features were extracted. A clinical-radiomics combined nomogram model for predicting LVI was constructed by integrating these features with clinical and laboratory indicators. Univariate and multivariate logistic regression analyses identified tumor volume, maximum tumor diameter, depth of tumor invasion, maximum short-axis diameter of regional lymph nodes, CEA level, neutrophil count, NLR, the SD of CT values within the tumor parenchyma, and the Rad-score as independent predictors of LVI positivity in CRC patients. The model validation results demonstrated an AUC of 0.776 in the training set and 0.722 in the validation set. The calibration curve indicated good agreement between the predicted probabilities and the actual LVI status.

As a core pathological feature reflecting the invasive and metastatic potential of CRC, the accurate preoperative prediction of LVI is crucial for optimizing treatment strategies (15, 16). In this study, NLR was identified as an independent predictor of LVI. NLR reflects the balance between systemic inflammatory response and anti-tumor immunity. An elevated NLR indicates a predominance of a neutrophil-mediated inflammatory microenvironment (17, 18). Neutrophils can facilitate tumor cell invasion by degrading the integrity of lymphatic and vascular walls through the release of substances such as matrix metalloproteinases and vascular endothelial growth factor (19).

The predictive value of the SD of CT values within the tumor parenchyma reflects intra-tumoral density heterogeneity (20). Tumor heterogeneity is a fundamental biological characteristic of malignancies. Greater density heterogeneity typically signifies significant variations in tumor cell proliferation activity, differentiation degree, and angiogenesis levels, which are associated with higher invasiveness and a greater propensity for lymphovascular invasion (21). The Rad-score, as a comprehensive quantitative metric integrating multiple radiomics features, was significantly associated with LVI prediction and demonstrated significant predictive value in the multivariate analysis. This finding aligns with the research approach of Li et al. in their CT-based radiomics study (10).

Furthermore, this study found that tumor volume, maximum diameter, depth of invasion, and the maximum short-axis diameter of regional lymph nodes were all correlated with LVI. Larger tumor volume and deeper invasion are associated with a higher probability of invasion into surrounding lymphatic and vascular structures (22). Enlargement of the regional lymph node short-axis diameter often suggests increased nodal burden, indirectly reflecting the tumor’s invasive and metastatic potential (23). The inclusion of CEA, a classical tumor marker whose elevation is associated with increased risk of tumor progression and metastasis (24), along with these other indicators, further enhanced the clinical relevance and predictive comprehensiveness of the model.

The model was developed using routine preoperative contrast-enhanced abdominal CT images from the portal venous phase. CT is a standard modality for preoperative evaluation of CRC, meaning this approach requires no additional imaging or healthcare costs. This confers excellent clinical accessibility and potential for broad adoption. The model integrates multi-dimensional information, including radiomics features, tumor morphological indicators, and laboratory results. Feature redundancy was addressed using LASSO regression, which improved model stability. This study adhered to sample size estimation principles, enrolling 360 patients, thereby meeting statistical requirements for model development.

This study has several limitations. First, it is a single-center retrospective study. Although the cohort was randomly divided into training and validation sets, external multi-center validation was not performed. The model’s generalizability may be influenced by geographic, population, and imaging equipment variations, necessitating further validation in subsequent multi-center studies. Second, the retrospective design inevitably introduces selection bias. Some potential confounding factors, such as the severity of patients’ underlying comorbidities, were not included in the analysis and may have a slight impact on the results. Third, region of interest (ROI) delineation relied on manual operation by physicians. Although reliability was ensured through dual-blinded delineation and consistency testing, a degree of subjective error remains. Future work could incorporate artificial intelligence-based automatic segmentation techniques to further reduce human interference. Fourth, this study did not incorporate tumor molecular biological characteristics, which may limit further improvement in the model’s predictive performance. Fifth, patients with a primary tumor maximum diameter of<5mm were excluded, thereby limiting the model’s applicability to very small lesions.

In clinical practice, for patients predicted by the model to be at high risk for LVI, more aggressive surgical strategies or preoperative neoadjuvant therapy could be considered to reduce the risk of postoperative recurrence and metastasis. For low-risk patients, overtreatment and its associated adverse effects and healthcare resource waste could be avoided, aligning with the principle of precision medicine. From a policy perspective, such prediction models based on routine imaging could be integrated into CRC diagnosis and treatment guidelines as decision-support tools, particularly valuable in primary healthcare settings lacking advanced imaging equipment.

Future research directions include the following: First, conducting multi-center, large-sample external validation to optimize model parameters and enhance its generalizability. Second, integrating tumor molecular biological characteristics with radiomics and clinical indicators to enrich the model’s dimensionality and improve predictive accuracy. Third, exploring the application of AI-based automatic segmentation in ROI delineation to reduce subjective error and increase application efficiency. Fourth, conducting dedicated studies on very small lesions to expand the model’s applicable scope.

In conclusion, the nomogram model developed in this study, which integrates radiomics signatures with clinical and imaging risk factors, can preoperatively predict LVI status in CRC patients with reasonable accuracy. Key predictive indicators include NLR, the SD of CT values within the tumor parenchyma, and the Rad-score. Based on routine preoperative CT, this model is operationally straightforward and highly accessible. It can provide a reference for clinical individualized treatment decision-making and prognosis assessment. However, external multi-center validation and further optimization are required to enhance its clinical application value.

## Data Availability

The original contributions presented in the study are included in the article/**Supplementary Material**. Further inquiries can be directed to the corresponding author.
