# Supplementary material for: A CT-based radiomics model for preoperative prediction of lymphovascular invasion in colorectal cancer
Source: Front Oncol. 2026 Apr 24;16:1811229. doi: 10.3389/fonc.2026.1811229 (PMC13152827; doi:10.3389/fonc.2026.1811229)
Supplement: Supplementary file 1 [file Table1.docx]

**Supplemental Table 1.** Univariate analysis of LVI status in CRC patients

| Variable | | Positive group (n=70) | Negative group (n=182) | *t/χ²* | *P* |
| --- | --- | --- | --- | --- | --- |
| Age (years) | | 58.25±7.52 | 56.88±8.12 | 1.224 | 0.222 |
| Gender | Male | 42(60.00) | 100(54.90) | 0.525 | 0.469 |
|  | Female | 28(40.00) | 82(45.10) |  |  |
| BMI (kg/m²) | | 23.45±2.33 | 23.81±2.32 | 1.102 | 0.272 |
| Smoking | Yes | 21(30.00) | 35(19.20) | 3.392 | 0.066 |
|  | No | 49(70.00) | 147(80.80) |  |  |
| Alcohol Drinking | Yes | 14(20.00) | 28(15.40) | 0.775 | 0.379 |
|  | No | 56(80.00) | 154(84.60) |  |  |
| Tumor Location | Colon | 28(40.00) | 80(43.90) | 0.323 | 0.570 |
|  | Rectum | 42(60.00) | 102(56.10) |  |  |
| CT-T | T1-2 | 12(17.14) | 35(19.23) | 0.319 | 0.853 |
|  | T3 | 45(64.29) | 110(60.44) |  |  |
|  | T4 | 13(18.57) | 37(20.33) |  |  |
| CT-N | N0 | 25(35.71) | 80(43.96) | 2.176 | 0.337 |
|  | N1 | 30(42.86) | 75(41.21) |  |  |
|  | N2 | 15(21.43) | 27(14.84) |  |  |
| Histological Grade | Well-differentiated | 8(11.43) | 25(13.74) | 0.247 | 0.884 |
|  | Moderately-differentiated | 45(64.29) | 115(63.19) |  |  |
|  | Poorly-differentiated | 17(24.29) | 42(23.08) |  |  |
| Tumor Volume (cm³) | | 9.78±4.51 | 8.82±3.25 | 1.874 | 0.062 |
| Maximum Tumor Diameter (mm) | | 35.15±8.22 | 32.42±7.55 | 2.515 | 0.013 |
| Distance from Anal Verge (cm) | | 7.55±3.02 | 8.22±4.04 | 1.258 | 0.210 |
| Tumor Invasion Depth (mm) | | 8.00±4.05 | 6.78±3.11 | 2.741 | 0.010 |
| Maximum Short-Axis Diameter of Regional Lymph Nodes (mm) | | 11.07±3.05 | 10.22±2.55 | 2.242 | 0.026 |
| CEA (ng/mL) | | 6.34±3.25 | 5.22±3.01 | 2.589 | 0.010 |
| CA19-9 (U/mL) | | 13.67±8.21 | 12.25±5.88 | 1.529 | 0.128 |
| CA125 (U/mL) | | 13.51±5.11 | 12.51±4.25 | 1.579 | 0.116 |
| NLR | | 2.45±1.14 | 2.12±0.88 | 2.447 | 0.015 |
| PLR | | 136.24±31.14 | 130.24±30.25 | 1.399 | 0.163 |
| Mean CT Value of Tumor Parenchyma (HU) | | 41.18±8.31 | 42.18±7.55 | 1.556 | 0.068 |
| Standard Deviation of CT Value of Tumor Parenchyma (HU) | | 9.84±3.22 | 8.85±2.52 | 2.577 | 0.011 |
| Rad-score | | 0.52±0.18 | 0.42±0.16 | 4.143 | 0.001 |
